# Agomelatine in the treatment of anhedonia, somatic symptoms, and sexual dysfunction in major depressive disorder

**DOI:** 10.3389/fpsyt.2023.1115008

**Published:** 2023-04-20

**Authors:** Juan Huang, Xiao-Meng Xie, Nan Lyu, Bing-Bing Fu, Qian Zhao, Ling Zhang, Gang Wang

**Affiliations:** ^1^Beijing Key Laboratory of Mental Disorders, National Clinical Research Center for Mental Disorders and National Center for Mental Disorders, Beijing Anding Hospital, Capital Medical University, Beijing, China; ^2^Advanced Innovation Center for Human Brain Protection, Capital Medical University, Beijing, China

**Keywords:** major depressive disorder, agomelatine, sexual dysfunction, anhedonia, somatic symptoms

## Abstract

**Objective:**

This study evaluated the treatment outcomes of agomelatine on anhedonic state, anxiety/somatic symptoms, and sexual function in Chinese patients with major depressive disorder (MDD).

**Method:**

In total, 93 adult patients with MDD were enrolled, and 68 of them were included in a prospective, open-label, multicenter clinical study. All patients received agomelatine monotherapy during a 9-week treatment phase. The effectiveness of the treatment was reflected by the improvement of anhedonia and somatic symptoms based on the 17-item Hamilton Depression Rating Scale (HAMD-17). In addition, the Arizona Sexual Dysfunction Scale (ASEX), Sheehan Disability Scale (SDS), and Short Form of Quality-of-Life Enjoyment and Satisfaction Questionnaire (Q-LES-Q-SF) were administered to all participants at baseline and at the 3-, 6-, and 9-week follow-ups.

**Results:**

After 9 weeks of treatment with agomelatine, the response and remission rates were 73.5% and 39.7%, respectively. Somatic symptoms significantly improved at week 9 (*p* < 0.001), and significant effects were also observed on the HAMD anhedonia items (*p* < 0.001). The patients exhibited lower levels of disease severity (the SDS score dropped from 15.52 ± 4.7 to 7.09 ± 5.62 at week 9; the ASEX score dropped from 21.89 ± 4.06 to 16.19 ± 4.79, *p* < 0.001) and higher levels of QOL (the Q-LES-Q-SF score dropped from 41.02 ± 5.99 to 50.49 ± 8.57, *p* < 0.001) during the follow-up. Furthermore, treatment with agomelatine improved depressive symptoms without causing serious adverse events.

**Conclusion:**

These analyses indicate that agomelatine is a treatment option for improving anhedonic status, anxiety/somatic symptoms, and sexual dysfunction in MDD patients.

## 1. Introduction

Major depressive disorder (MDD) is one of the most prevalent, burdensome, and costly psychiatric disorders in adults globally (1). MDD is associated with significant functional impairment and high morbidity and mortality (2). The World Health Organization (WHO) has reported that MDD is the leading cause of disability in the world (3). Although both pharmacological and non-pharmacological interventions are available for MDD treatment, antidepressants with different mechanisms are a milestone in treatment progress (4). Fully functional recovery, which is the ultimate treatment goal for patients with MDD, may be unsuccessful in some patients. The Sequenced Treatment Alternatives to Relieve Depression (STAR^*^D) study reported that up to 30% of patients with MDD fail to achieve adequate remission (5). Different subtypes or presented symptoms of MDD partly account for discrepant treatment outcomes, functioning, and quality of life (QOL) (6, 7). Similarly, somatic and residual symptoms are also the most common risk factors for therapeutic inefficiency (8) in MDD patients.

Anhedonia is a predictor of poor treatment response in patients with MDD (9, 10). Anhedonia, the diminished interest and ability to experience pleasure, is conceptualized as a core feature of MDD (11). Anhedonia is difficult to treat, as accumulated evidence has shown that current first-line antidepressant treatments [such as selective serotonin reuptake inhibitors (SSRIs)] have limited effectiveness in treating defects in motivation and reward processing (12–14).

In clinical practice, somatic symptoms usually present as the major complaints of MDD (15–17). Anxiety/somatic symptoms are characterized by anxiety and worries that are difficult to control and by accompanying psychic and somatic symptoms, including sleep disturbance (18). Previous literature has shown that patients with MDD in Asian countries, especially in China, are more likely to emphasize their somatic symptoms instead of other depressive symptoms than their counterparts in Western countries (19, 20). In addition to somatic complaints, sexual dysfunction is also commonly associated with poor medication adherence (21), a higher incidence of relapse (22), and a negative impact on quality of life (23). Therefore, the therapeutic care of this population with an anhedonic state, somatic symptoms, and sexual dysfunction raises particular clinical concerns.

Agomelatine is an antagonist targeting the postsynaptic serotonin receptor 5-HT2c and melatonergic receptor agonist (MT1/MT2). Agomelatine has been approved for the treatment of MDD (24, 25). As the first approved drug targeting the melatoninergic system rather than the monoaminergic system (26), the efficacy and safety of agomelatine in treating MDD have been established in several randomized controlled trials with placebo or active controls (18, 27, 28). In a 12-week double-blind comparison study, favorable effects of agomelatine were shown in many psychopathological conditions, extending beyond emotional symptoms (29). Published articles have shown that agomelatine is beneficial for sleep structure; it resynchronizes the sleep–wake cycle by acting on melatonin receptors. Additionally, agomelatine has anti-anxiety effects along with antidepressant properties (30).

Previous RCTs have shown that agomelatine seems to be an efficacious antidepressant for treatment-resistant depression or residual symptoms of depression. However, there are limited studies about agomelatine on the issues stated above for treatment in the Chinese population (31, 32). Therefore, a prospective, multicenter, and interventional study was conducted to confirm the effectiveness and safety of agomelatine in treating anhedonia, depression-related somatic symptoms, and sexual dysfunction in patients with MDD.

## 2. Methods

### 2.1. Setting and participants

The project was a multicenter 9-week interventional study conducted at three mental healthcare centers in China located in Beijing, Jinan, and Harbin. The recruitment of this project lasted for 1 year. Patients with MDD who fulfilled the following inclusion and exclusion criteria were eligible for participation in this trial.

The eligibility criteria were patients who (1) were aged from 18 to 65 years; (2) were diagnosed with MDD according to the Diagnostic and Statistical Manual of Mental Disorders, Fourth Edition, Text Revision (DSM-IV-TR); (3) were comorbid with non-psychotic symptoms; (4) had a total score of ≥17 on the 17-item Hamilton Depression Rating Scale (HAMD-17); (5) were able to communicate in Chinese; and (6) provided written informed consent.

Patients were excluded from the trial if they (1) were currently or previously diagnosed with any other psychiatric disorders other than MDD; (2) had a serious and unstable medical or surgical condition; (3) were a hepatitis B carrier or had a history of liver disease or hepatic and renal failure; (4) suffered from abuse/dependence on alcohol or other substances; (5) previously did not fully respond to systemic treatment of agomelatine; (6) presented obvious suicide attempt or behavior; (7) were hypersensitive to agomelatine or the excipients; (8) were pregnant or lactating; (9) participated in systemic psychotherapeutic therapies or electroconvulsive therapy during the recent 3 months; (10) used monoamine oxidase inhibitors (MAOIs) in the recent 2 weeks or fluoxetine in the recent month; or (11) participated in other clinical trials within the month.

The acute phase of depression requires 6–12 weeks of treatment. Combined with the current registration protocols of agomelatine studies (18, 33) and considering the safety requirements of the drug instructions, we chose to extend the visit for 1 week on the basis of 8 weeks to better observe the side effects. The clinical trial was registered in a public trials registry to be considered for publication (ID: ChiCTR2200066866), and the authors were compliant with the Consolidated Standards of Reporting Trials (CONSORT).

### 2.2. Treatment

All participants were asked to orally take a 25 mg/days dose of agomelatine before sleep. The dose could be increased to 25–50 mg/days as determined by their treating psychiatrists, who were also researchers in this study. The increased dose was based on the assessment of tolerability and clinical response at 3 weeks of treatment, and this dose remained constant until the end of the trial. Mood stabilizers, antipsychotics, and antidepressants other than agomelatine were not allowed during the trial. Temporary use of anti-anxiety drugs (e.g., short-acting non-benzodiazepines) was permitted to relieve insomnia.

### 2.3. Outcome assessments

Basic sociodemographic and clinical variables, such as duration of current episode years, gender, age, marital status, educational level (illiterate/primary/secondary school education *vs*. college education and above), occupational status, history of previous physical disorders, drug therapy for somatic diseases in the past 6 months, experience of anti-psychotherapy in the past 6 months, and family history of psychosis, were collected. The Chinese version of the HAMD-17 was applied to assess the severity of depression, which is the primary outcome of this study. The HAMD-17 has been validated in the Chinese population with a sensitivity of 0.85 and a specificity of 0.92 (34). Referring to previous studies (35), the total score of items assessing anxiety/somatic symptoms in the HAMD-17 (Items 10, 11, 12, 13, 15, and 17) was used to evaluate the severity of anxiety/somatic symptoms in this study, while the score of Item 7 of the HAMD-17 was used to measure the severity of anhedonia (36, 37). The effectiveness outcomes are as follows: The Arizona Sexual Dysfunction Scale (ASEX), which quantifies the patient's sex drive, arousal, vaginal lubrication/penile erection, ability to reach orgasm, and satisfaction from orgasm, was used to assess sexual dysfunction, with a higher total score indicating more severe sexual dysfunction (38). Cronbach's alpha coefficient of the Chinese ASEX was 0.831 (39). The Sheehan Disability Scale (SDS) was used to assess general function (i.e., three functional domains: work/school, social life, and family life or home responsibilities) (40), and the internal consistency Cronbach's alpha for the total SDS-C score was 0.94 (40). Quality of life (QOL) was evaluated using the Short Form of Quality-of-Life Enjoyment and Satisfaction Questionnaire (Q-LES-Q-SF) (41). Each item of the Q-LES-Q-SF was scored from 0 to 5, and a higher total score indicated better QOL. The Chinese version of the scale has been validated with satisfactory psychometric properties (42). Early change in score compared to baseline and scores were collected at each visit (weeks 3, 6, and 9) by clinical study investigators.

The response was defined as a ≥50% reduction in the HAMD-17 total score at the endpoint assessment compared to the baseline assessment. Remission was defined as a HAMD-17 total score ≤ 7 at the endpoint assessment (43). The safety of agomelatine monotherapy was detected in the following aspects at each assessment point: treatment-emergent adverse events (TEAEs), body weight, blood pressure, heart rate, and laboratory examinations.

### 2.4. Ethical aspects

The study was conducted in accordance with the current version of the Declaration of Helsinki. The protocol was approved by the participating hospitals' Ethics Committee (No. (2017) (78)-201803FS-2).

### 2.5. Data analysis

The data were analyzed using SAS^®^ (SAS Institute Inc., NC, USA). The dropout rates of nearly 20% of overall items of instruments were considered missing data. The descriptive statistics for continuous variables consisted of the mean and standard deviation. Categorical variables were described as frequencies and proportions, and a 95% confidence interval (95% CI) was appropriate. The mixed model for repeated-measures analysis of each visit was performed to account for the multiple assessments obtained during this study. Comparison of effectiveness assessments' change from baseline to each evaluation point was conducted using the least squares mean pairwise comparison, performed by Dunnett's *t*-test. If convergence failed, stimulate adjustment was adopted. The correlation was analyzed with a *p-*value of <0.05 considered statistically significant (two-tailed).

## 3. Results

**Figure 1:**
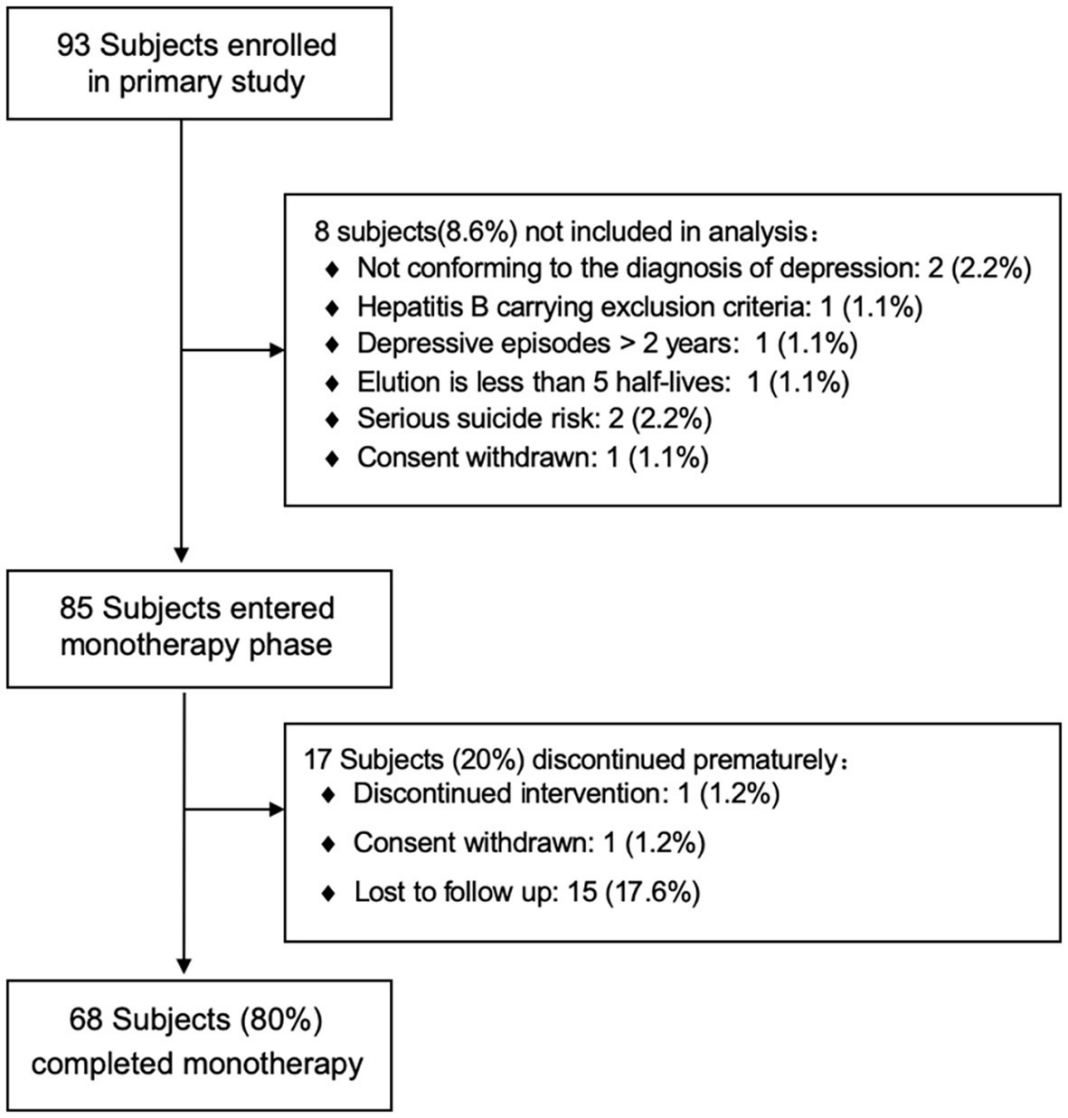
Flow chart of the study procedure.

A total of 93 patients participated in this study. Eighty-five patients (25 men and 60 women) with a mean age of 40.11 years [standard deviation (SD) = 13.71] met the inclusion criteria and accepted agomelatine monotherapy (Figure 1). Seventeen patients dropped out of the trial, 15 of whom were lost to follow-up, one discontinued agomelatine, and one withdrew informed consent. Ultimately, 68 patients completed the 9-week trial and were included in the analysis. The baseline demographics and clinical characteristics of the subjects are summarized in Table 1. The average doses of agomelatine at weeks 3, 6, and 9 were 35.47 (SD = 12.42) mg, 40.14 (SD = 12.30) mg, and 41.04 (SD = 12.07) mg, respectively.

**Table 1:** Demographic characteristics of patients completing the trial at baseline (*N* = 68).

**Variables**	***N***	**%**
**Gender**		
Female	46	68.7
Male	22	31.3
**Marital status**		
Single	10	14.7
Married	41	60.3
Divorced/separated/widowed	17	25.0
**Current occupational status**		
Full-time job	35	51.5
Part-time job	23	33.8
Unemployed	10	14.7
**Educational background**		
Illiterate	1	1.5
Primary school education	10	14.7
Secondary school education	27	39.7
College education and above	30	44.1
History of previous physical disorders	26	38.2
Drug therapy for somatic diseases in the past 6 months	11	16.1
Experience of anti-psychotherapy in the past 6 months	12	17.6
Family history of psychosis	12	17.6
	**Mean**	**SD**
Age years	42.1	13.4
Duration of current episode years	0.3	0.8
Agomelatine mean dose (mg)	25	0
**At baseline, total score of**		
HAMD-17 total	26.6	6.5
HAMD-17 anxiety/somatic symptoms^#^	7.2	2.0
HAMD-17 anhedonia^*^	2.6	0.06
Q-LES-Q-SF	41.0	6.0
ASEX	21.9	4.1
SDS	15.5	4.7

# Items 10, 11, 12, 13, 15, and 17 of HAMD-17.

* Item 7 of HAMD-17.

SD, standard deviation; HAMD-17, 17-item Hamilton Depression Rating Scale; Q-LES-Q-SF, quality-of-life enjoyment and satisfaction questionnaire-short form; ASEX, Arizona sexual experiences scale; SDS, Sheehan disability scale.

The HAMD-17 total score significantly decreased as early as the third week of treatment (*p* < 0.001). The response rate was 73.5%; 39.7% of the patients achieved remission.

The total score of the HAMD-17 anxiety/somatic symptoms subscale significantly decreased as early as the third week of treatment (*p* < 0.001) and significantly decreased from 6.95 (SD = 2.12) at baseline to 2.16 (SD = 2.03) at the endpoint of the trial (*p* < 0.001). The mixed-effect model revealed that there was no significant difference in the change in the anxiety/somatic symptoms subscale score among the three study sites. Temporal and multicenter interactions were significant (*F* = 11.81, *p* < 0.001).

**Figure 2:**
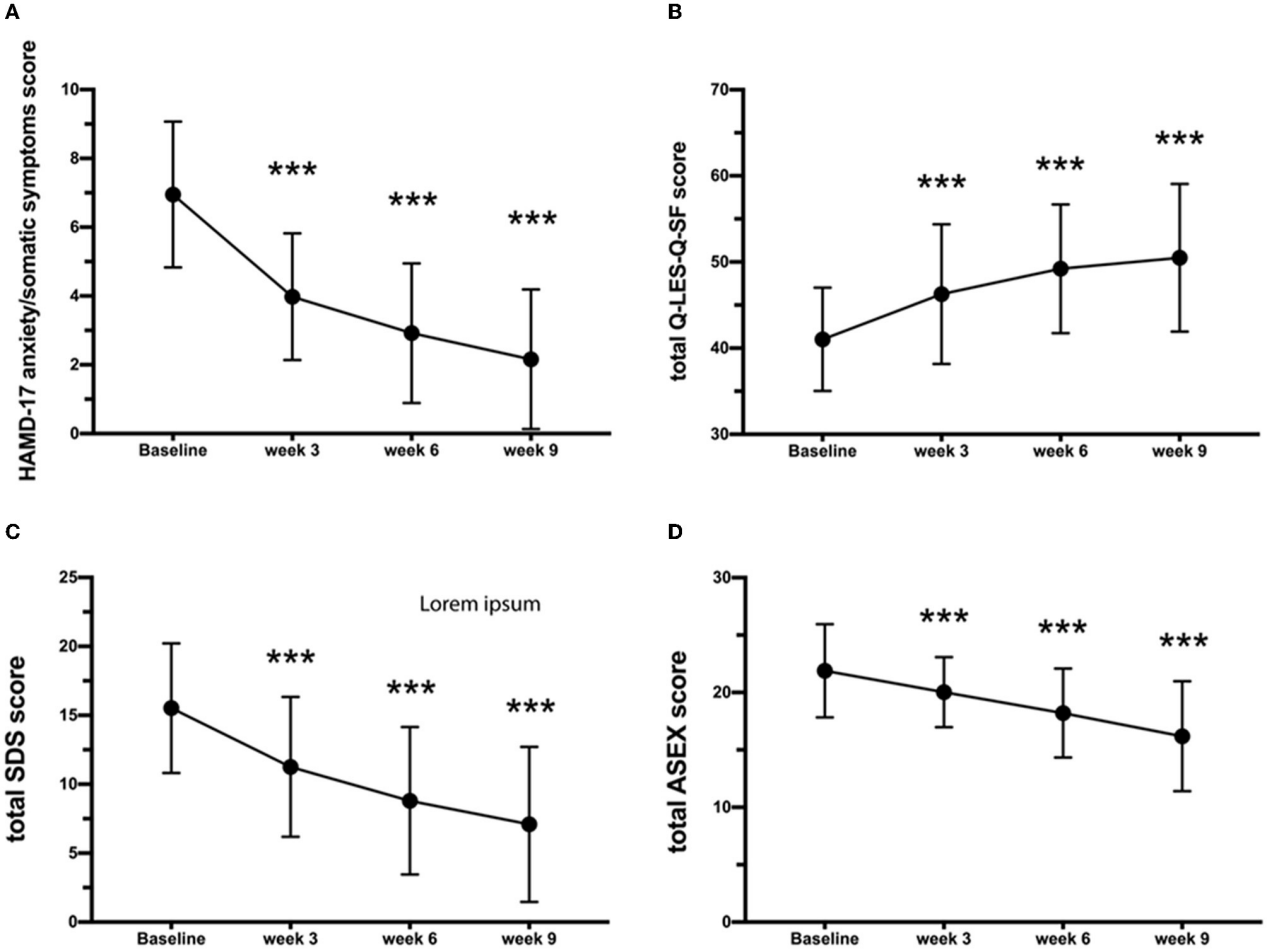
Variations in **(A)** HAMD-17 anxiety/somatic, **(B)** Q-LES-Q-SF, **(C)** SDS, and **(D)** ASEX scores. Results are expressed as mean ± SD, analysis of variance for repeated measures. ***Statistically significant difference from baseline (*P* < 0.001). Error bars represent standard deviations. HAMD-17, 17-item Hamilton Depression Rating Scale; QIDS-SR, quick inventory of depressive symptomatology self-report; CGI-S, clinical global impression scale; Q-LES-Q-SF, quality-of-life enjoyment and satisfaction questionnaire-short form; ASEX, Arizona sexual experiences scale; SDS, Sheehan Disability Scale.

The scores of anhedonia were 2.58 (SD = 0.06), 1.68 (SD = 0.09), 1.07 (SD = 0.09), and 0.93 (SD = 0.09) at baseline and weeks 3, 6, and 9 of treatment, respectively. A significant reduction in the severity of anhedonia was observed from the second follow-up (*p* < 0.001) until the endpoint of the trial. About effectiveness outcomes, the Q-LES-Q-SF total score significantly increased from 41.02 (SD = 5.99) at baseline to 46.27 (SD = 8.11) at week 3 (*p* < 0.001) and 50.49 (SD = 8.57) at week 9 (*p* < 0.001) (Figure 2). There were no significant differences in the change in the Q-LES-Q-SF total score across the three study sites. Temporal and multicenter interactions were significant (*F* = 7.36, *p* < 0.001). Both the total scores of the ASEX and SDS significantly decreased from the baseline [ASEX: 21.89 (SD = 4.06); SDS: 15.52 (SD = 4.7), *p* < 0.001] to the week 3 treatment [ASEX: 20.03 (SD = 3.04); SDS: 11.26 (SD = 5.07), *p* < 0.001] and to the week 9 treatment [ASEX: 16.19 (SD = 4.79); SDS: 7.09 (SD = 5.62), *p* < 0.001, Figure 2]. Temporal and multicenter are significant in the mixed-effect model (ASEX: *F* = 25.64, *p* < 0.001; SDS: *F* = 15.65, *p* < 0.001). A total of 17 adverse events were spontaneously reported. The most frequently reported adverse events were insomnia (10.29%) and agitation (8.82%). After 6 weeks of treatment, one case (1.47%) reported elevation of serum concentration of aspartate aminotransferase (AST) and alanine aminotransferase (ALT) but recovered at week 9 without any additional liver protection treatment. Three events, including influenza (1.47%), nasopharyngitis (1.47%), and constipation (1.47%), were determined to be unrelated to agomelatine. Another 14 patients showed abnormalities in clinical tests and vital signs after treatment, such as elevated total bilirubin (TBil), elevated uric acid (UA), and abnormal blood pressure, but none were serious or related to the laboratory test values.

## 4. Discussion

To the best of our knowledge, this was the first prospective, multicenter study to examine the effectiveness outcomes of agomelatine on anxiety/somatic symptoms, anhedonia, and sexual dysfunction in patients with MDD in China. After 9 weeks of treatment, the response rate was 73.5%, and the remission rate was 39.7%. Gargoloff et al. (44) defined remission as a score of ≤ 5 on the QIDS-SR-16. They reported that the remission rate of agomelatine was 49.6% when treating MDD outpatients. The corresponding response rate was 65.7%. The daily dose of agomelatine in Gargoloff's study was 25–50 mg, which was comparable to the average daily dose of agomelatine in our study (41.04 mg, SD = 12.07 mg). Meanwhile, a network meta-analysis showed that MDD patients treated with agomelatine and venlafaxine had higher remission rates (RR = 1.36; 95% CI = 1.05–1.76) than those treated with fluoxetine and sertraline (27).

This study found that agomelatine produced a significant improvement in anxiety/somatic symptoms as early as the third week of treatment. This positive effect on anxiety/somatic symptoms is consistent with a previous report on agomelatine (25–50 mg/day) vs. escitalopram (10–20 mg/day) in outpatient treatments. The psychic and somatic anxiety scores clinically significantly decreased, alertness and sleep parameters improved, and ability to experience pleasure increased, using the HAMA scale (18). Another narrative review investigating the efficacy of agomelatine on individual HAMD items found that agomelatine significantly outperformed placebo in 10 items, including psychic and somatic anxiety, as well as somatic symptoms, suggesting that agomelatine is effective for a broad spectrum of depressive symptoms (45). In an 8-week trial comparing the efficacy of agomelatine and fluoxetine when treating MDD, both of these antidepressants were effective in reducing the HAMA somatic anxiety subscale score; however, the change in this subscale score had no significant difference between the two treatment groups (46). Previous studies have suggested that fluoxetine has beneficial effects on specific anxiety disorders (e.g., generalized anxiety disorder, social anxiety disorder, and panic disorder) (47–49). More head-to-head studies with comparable conditions are needed to provide more convincing evidence. However, agomelatine has more rapid action on dendritic maturation than selective serotonin reuptake inhibitors (SSRIs) and any other monoaminergic antidepressant (50), which has also been demonstrated in animal models of depression/anxiety (51). The actions of agomelatine on dendritic maturation are thought to be mediated through both MT and 5-HT_2C_ receptors present in the ventral hippocampus, which are involved in the emotional circuitry controlling depressive/anxiety states (52, 53).

As a core symptom of MDD, anhedonia is considered crucial in treatment and as a potential trait marker related to vulnerability to illness relapse (54, 55). In this study, agomelatine significantly improved anhedonia from the third week of treatment (*P* < 0.001). This finding is consistent with previous research that agomelatine produced, as early as the first week following treatment initiation, a significant improvement in anhedonia measured by the Snaith–Hamilton Pleasure Scale (SHAPS) (56). A pilot 8-week trial reported that agomelatine showed significantly better effectiveness when treating anhedonia in MDD compared to venlafaxine as assessed by the SHAPS (57). The pharmacological effects of enhancing dopamine and noradrenaline transmission, as well as agonist actions at melatonin receptors in the prefrontal cortex, were thought to be responsible for the effect of agomelatine in treating anhedonia (25, 58).

Moreover, due to the function of agomelatine in specifically limbic areas without influencing extracellular serotonin (5-HT) levels (56, 59), agomelatine is well tolerated and associated with lower levels of sexual dysfunction, gastrointestinal reactions, headaches, weight gain, daytime somnolence, and serotonin syndrome, which would have important advantages. These characteristics are important advantages that are different from SSRIs. Our results support previous findings that agomelatine is associated with a low incidence of sexual dysfunction. In several randomized, placebo/active control trials, agomelatine appeared to consistently have a lower propensity to cause sexual dysfunction (60–63). Agomelatine was associated with less decreased desire (agomelatine 6% vs. venlafaxine 16.4%; *p* < 0.0001), less orgasmic disorder (agomelatine 9.1% vs. venlafaxine 18.5%; *p* = 0.001), and better overall sexual function (agomelatine 8.2% vs. venlafaxine 15.2%; *p* < 0.0001) in both genders (60).

Agomelatine monotherapy has satisfactory effectiveness and tolerance, especially for improving somatic symptoms, sexual dysfunction, and insomnia in previous studies (64–66), which is helpful for the comprehensive rehabilitation of patients with MDD. A network meta-analysis on the acceptability of 21 antidepressants for the treatment of patients with MDD at an acute illness stage found that agomelatine, citalopram, escitalopram, fluoxetine, sertraline, and vortioxetine were more acceptable than other antidepressants (range of ORs = 0.43–0.77) (31). In this study, 17 cases of mild-to-moderate adverse events were reported, and 14 cases of abnormal laboratory results with no clinical significance were reported. The incidence of adverse events was consistent with a previous study, which found that in a 6-month extension trial, compared to venlafaxine and sertraline, the percentage of patients who discontinued treatment due to adverse events was lower in those treated with agomelatine (agomelatine 4.2% vs. venlafaxine 14.9%, *p* = 0.001; agomelatine 6.7% vs. 12.5% for sertraline 12.5%, *p* = 0.09) (67).

Several limitations should be noted. First, the effectiveness results are strictly exploratory and thus should be interpreted with caution due to a lack of placebo control. Second, due to logistical reasons, relevant factors related to the effectiveness of agomelatine, such as the duration of illness or the number (and duration) of previous depressive episodes, lifestyle, family support, and medication adherence, which may influence the current response to antidepressants, were not obtained. Third, our data on sexual dysfunction were collected from self-report questionnaires, and different results might have been obtained with diagnostic interviews. Fourth, because this was a study in the real world, convenience sampling was used, and gender biases were inevitable; therefore, the number of participants should be increased in the future. Finally, the number of participants was limited, so a large number of participants along with random controlled studies are needed in the future.

To summarize, current evidence indicates that MDD patients treated with agomelatine at a daily dose of 25–50 mg had satisfactory treatment outcomes regarding remission rate, response rate, and the severity of anhedonia, somatic symptoms, and sexual dysfunction. This study also provides further evidence that a daily dose of 25–50 mg of agomelatine is safe and tolerable. These findings are important to improve the treatment strategy for MDD to provide broad symptom relief and achieve complete functional recovery. Agomelatine not only improves anhedonia and sexual dysfunction but also relieves somatic symptoms. More clinical trials on the global effectiveness of agomelatine are warranted to confirm the present findings.

## Data availability statement

There are stringent restrictions in making the research dataset of the clinical studies publicly available. Readers and all interested researchers may contact LZ (Email address: zhangling@ccmu.edu.cn) to apply for exemptions from the participating institutions if appropriate.

## Ethics statement

The studies involving human participants were reviewed and approved by Anding hospitals' Ethics Committee. The patients/participants provided their written informed consent to participate in this study.

## Author contributions

GW and LZ: study design. JH, X-MX, and NL: collection, analyses, and interpretation of data. JH, X-MX, B-BF, and QZ: drafting of the manuscript. GW: critical revision of the manuscript. All authors approved the final version for publication.

## References

[1] Auerbach RP Stanton CH Proudfit GH Pizzagalli DA. Self-referential processing in depressed adolescents: a high-density event-related potential study. J Abnorm Psychol. (2015) 124:233–45. 10.1037/abn0000023 25643205 PMC4429006

[2] Michaud CM Murray CJ Bloom BR. Burden of disease: implications for future research. JAMA. (2001) 285:535–9. 10.1001/jama.285.5.535 11176854

[3] World Health Organization. (2021). Depression. Geneva: World Health Organization. Available online at: https://www.who.int/health-topics/depression#tab=tab/1 (accessed Jan 13, 2021).

[4] Ioannidis JP. Effectiveness of antidepressants: an evidence myth constructed from a thousand randomized trials? Philos Ethics Humanit Med. (2008) 3:14. 10.1186/1747-5341-3-14 18505564 PMC2412901

[5] Rush AJ Trivedi MH Wisniewski SR Nierenberg AA Stewart JW Warden D. Acute and longer-term outcomes in depressed outpatients requiring one or several treatment steps: a STAR^*^D report. Am J Psychiatry. (2006) 163:1905–17. 10.1176/ajp.2006.163.11.1905 17074942

[6] Arnow BA Hunkeler EM Blasey CM Lee J Constantino MJ Fireman B. Comorbid depression, chronic pain, and disability in primary care. Psychosom Med. (2006) 68:262–8. 10.1097/01.psy.0000204851.15499.fc 16554392

[7] Fried EI Nesse RM Zivin K Guille C Sen S. Depression is more than the sum score of its parts: individual DSM symptoms have different risk factors. Psychol Med. (2014) 44:2067–76. 10.1017/S0033291713002900 24289852 PMC4104249

[8] Nierenberg AA. Residual symptoms in depression: prevalence and impact. J Clin Psychiatry. (2015) 76:e1480. 10.4088/JCP.13097TX1C 26646047

[9] Gao K Sweet J Su M Calabrese JR. Depression severity and quality of life of qualified and unqualified patients with a mood disorder for a research study targeting anhedonia in a clinical sample. Asian J Psychiatr. (2017) 27:40–7. 10.1016/j.ajp.2017.02.013 28558894

[10] Spijker J Bijl R De Graaf R Nolen W. Determinants of poor 1-year outcome of DSM-III-R major depression in the general population: results of the Netherlands mental health survey and incidence study (NEMESIS). Acta Psych Scand. (2001) 103:122–30. 10.1034/j.1600-0447.2001.103002122.x 11167315

[11] Treadway MT Zald DH. Reconsidering anhedonia in depression: Lessons from translational neuroscience. Neurosci Biobehav Rev. (2011) 35:537–55. 10.1016/j.neubiorev.2010.06.006 20603146 PMC3005986

[12] McCabe C Mishor Z Cowen PJ Harmer CJ. Diminished neural processing of aversive and rewarding stimuli during selective serotonin reuptake inhibitor treatment. Biol Psychiatry. (2010) 67:439–45. 10.1016/j.biopsych.2009.11.001 20034615 PMC2828549

[13] Price J Cole V Goodwin GM. Emotional side-effects of selective serotonin reuptake inhibitors: qualitative study. Br J Psychiatry. (2009) 195:211–7. 10.1192/bjp.bp.108.051110 19721109

[14] Shelton RC Tomarken AJ. Can recovery from depression be achieved? Psych Serv. (2001) 52:1469–78. 10.1176/appi.ps.52.11.1469 11684742

[15] Lin CF Juang YY Wen JK Liu CY Hung CI. Correlations between sexual dysfunction, depression, anxiety, and somatic symptoms among patients with major depressive disorder. Chang Gung Med J. (2012) 35:323–31. 10.4103/2319-4170.106138 22913859

[16] Mathew RJ Weinman ML Thapar R Reck JJ Claghorn JL. Somatic symptoms in depression and antidepressants. J Clin Psychiatry. (1983) 44:10–2. 6822481

[17] Nelson JC Portera L Leon AC. Residual symptoms in depressed patients after treatment with fluoxetine or reboxetine. J Clin Psychiatry. (2005) 66:1409–14. 10.4088/JCP.v66n1110 16420078

[18] Stein DJ Khoo JP Ahokas A Jarema M Van Ameringen M Vavrusova L. 12-week double-blind randomized multicenter study of efficacy and safety of agomelatine (25–50 mg/day) vs. escitalopram (10–20 mg/day) in out-patients with severe generalized anxiety disorder. Eur Neuropsychopharmacol. (2018) 28:970–9. 10.1016/j.euroneuro.2018.05.006 30135032

[19] Novick D Montgomery W Aguado J Kadziola Z Peng X Brugnoli R. Which somatic symptoms are associated with an unfavorable course in Asian patients with major depressive disorder? J Affect Disord. (2013) 149:182–8. 10.1016/j.jad.2013.01.020 23521872

[20] Ryder AG Yang J Zhu X Yao S Yi J Heine SJ. The cultural shaping of depression: somatic symptoms in China, psychological symptoms in North America? J Abnorm Psychol. (2008) 117:300. 10.1037/0021-843X.117.2.300 18489206

[21] Serrano MJ Vives M Mateu C Vicens C Molina R Puebla-Guedea M. Therapeutic adherence in primary care depressed patients: a longitudinal study. Actas Esp Psiquiatr. (2014) 42:91–8. 24844808

[22] Silverstone PH Salinas E. Efficacy of venlafaxine extended release in patients with major depressive disorder and comorbid generalized anxiety disorder. J Clin Psychiatry. (2001) 62:523–9. 10.4088/JCP.v62n07a04 11488362

[23] Piontek A Szeja J Błachut M Badura-Brzoza K. Sexual problems in the patients with psychiatric disorders. Wiad Lek. (2019) 72:1984–8. 10.36740/WLek201910125 31982027

[24] Masson-Pévet M Recio J Guerrero HY Mocaer E Delagrange P Guardiola-Lemaitre B. Effects of two melatonin analogues, S-20098 and S-20928, on melatonin receptors in the pars tuberalis of the rat. J Pineal Res. (1998) 25:172–6. 9745986 10.1111/j.1600-079x.1998.tb00556.x

[25] Millan MJ Gobert A Lejeune F Dekeyne A Newman-Tancredi A Pasteau V. The novel melatonin agonist agomelatine (S20098) is an antagonist at 5-hydroxytryptamine2C receptors, blockade of which enhances the activity of frontocortical dopaminergic and adrenergic pathways. J Pharmacol Exp Ther. (2003) 306:954–64. 10.1124/jpet.103.051797 12750432

[26] Gorwood P Benichou J Moore N Wattez M Secouard MC Desobry X. Agomelatine in standard medical practice in depressed patients: results of a 1-year multicentre observational study in France. Clin Drug Investig. (2020) 40:1009–20. 10.1007/s40261-020-00957-9 32729068 PMC7595961

[27] Khoo AL Zhou HJ Teng M Lin L Zhao YJ Soh LB. Network meta-analysis and cost-effectiveness analysis of new generation antidepressants. CNS Drugs. (2015) 29:695–712. 10.1007/s40263-015-0267-6 26293743

[28] Singh SP Singh V Kar N. Efficacy of agomelatine in major depressive disorder: meta-analysis and appraisal. Int J Neuropsychopharmacol. (2012) 15:417–28. 10.1017/S1461145711001301 21859514

[29] Montgomery SA Kennedy SH Burrows GD Lejoyeux M Hindmarch I. Absence of discontinuation symptoms with agomelatine and occurrence of discontinuation symptoms with paroxetine: a randomized, double-blind, placebo-controlled discontinuation study. Int Clin Psychopharmacol. (2004) 19:271–80. 10.1097/01.yic.0000137184.64610.c8 15289700

[30] Naveed M Li LD Sheng G Du ZW Zhou YP Nan S. Agomelatine: an astounding sui-generis antidepressant? Curr Mol Pharmacol. (2022) 15:943–61. 10.2174/1874467214666211209142546 34886787

[31] Cipriani A Furukawa TA Salanti G Chaimani A Atkinson LZ Ogawa Y. Comparative efficacy and acceptability of 21 antidepressant drugs for the acute treatment of adults with major depressive disorder: a systematic review and network meta-analysis. Lancet. (2018) 391:1357–66. 10.1016/S0140-6736(17)32802-7 29477251 PMC5889788

[32] Goodwin GM Emsley R Rembry S Rouillon F. Agomelatine prevents relapse in patients with major depressive disorder without evidence of a discontinuation syndrome: a 24-week randomized, double-blind, placebo-controlled trial. J Clin Psychiatry. (2009) 70:1128–37. 10.4088/JCP.08m04548 19689920

[33] Yu YM Gao KR Yu H Shen YF Li HF. Efficacy and safety of agomelatine vs. paroxetine hydrochloride in Chinese Han patients with major depressive disorder: a multicentre, double-blind, non-inferiority, randomized controlled trial. J Clin Psychopharmacol. (2018) 38:226–33. 10.1097/JCP.0000000000000878 29620692

[34] Zheng YP Zhao JP Phillips M Liu JB Cai MF Sun SQ. Validity and reliability of the Chinese Hamilton Depression Rating Scale. Br J Psychiatry. (1988) 152:660–4. 3167442 10.1192/bjp.152.5.660

[35] Farabaugh A Mischoulon D Fava M Wu SL Mascarini A Tossani E. The relationship between early changes in the HAMD-17 anxiety/somatization factor items and treatment outcome among depressed outpatients. Int Clin Psychopharmacol. (2005) 20:87–91. 10.1097/00004850-200503000-00004 15729083

[36] Dombrovski AY Cyranowski JM Mulsant BH Houck PR Buysse DJ Andreescu C. Which symptoms predict recurrence of depression in women treated with maintenance interpersonal psychotherapy? Depress Anxiety. (2008) 25:1060–6. 10.1002/da.20467 18781665 PMC2705944

[37] Tang W Liu H Chen L Zhao K Zhang Y Zheng K. Inflammatory cytokines, complement factor H and anhedonia in drug-naïve major depressive disorder. Brain Behav Immun. (2021) 95:238–44. 10.1016/j.bbi.2021.03.022 33794316

[38] McGahuey A Gelenberg AJ Laukes CA Moreno FA Delgado PL McKnight KM. The arizona sexual experience scale (ASEX): reliability and validity. J Sex Marital Therapy. (2000) 26:25–40. 10.1080/009262300278623 10693114

[39] Zhu RX Hou G. The influence of antipsychotic treatment on sexual function in remitted male schizophrenic patients and the correlation with plasma prolactin concentrations (in Chinese). J Clin Psychiatry. (2010) 20:322–3.

[40] Leu SH Chou JY Lee PC Cheng HC Shao WC Hsien WL. Validity and reliability of the Chinese version of the Sheehan disability scale (SDS-C). Asia Pac Psychiatry. (2015) 7:215–22. 10.1111/appy.12182 25847187

[41] Endicott J Paulsson B Gustafsson U Schiöler H Hassan M. Quetiapine monotherapy in the treatment of depressive episodes of bipolar I and II disorder: improvements in quality of life and quality of sleep. J Affect Disord. (2008) 111:306–19. 10.1016/j.jad.2008.06.019 18774180

[42] Lee YT Liu SI Huang HC Sun FJ Huang CR Yeung A. Validity and reliability of the Chinese version of the short form of quality of life enjoyment and satisfaction questionnaire (Q-LES-Q-SF). Qual Life Res. (2014) 23:907–16. 10.1007/s11136-013-0528-0 24062242

[43] Trivedi MH Rush AJ Wisniewski SR Nierenberg AA Warden D Ritz L. Evaluation of outcomes with citalopram for depression using measurement-based care in STAR^*^ D: implications for clinical practice. Am J Psychiatry. (2006) 163:28–40. 10.1176/appi.ajp.163.1.28 16390886

[44] Gargoloff PD Corral R Herbst L Marquez M Martinotti G Gargoloff PR. Effectiveness of agomelatine on anhedonia in depressed patients: an outpatient, open-label, real-world study. Hum Psychopharmacol. (2016) 31:412–8. 10.1002/hup.2557 27859669

[45] Demyttenaere K. Agomelatine: a narrative review. Eur Neuropsychopharmacol. (2011) 21:S703–709. 10.1016/j.euroneuro.2011.07.004 21835598

[46] Hale A Corral RM Mencacci C Ruiz JS Severo CA Gentil V. Superior antidepressant efficacy results of agomelatine vs. fluoxetine in severe MDD patients: a randomized, double-blind study. Int Clin Psychopharmacol. (2010) 25:305–14. 10.1097/YIC.0b013e32833a86aa 20856123

[47] Dulawa SC Holick KA Gundersen B Hen R. Effects of chronic fluoxetine in animal models of anxiety and depression. Neuropsychopharmacology. (2004) 29:1321–30. 10.1038/sj.npp.1300433 15085085

[48] Sonawalla SB Farabaugh A Johnson MW Morray M Delgado ML Pingol MG. Fluoxetine treatment of depressed patients with comorbid anxiety disorders. J Psychopharmacol. (2002) 16:215–9. 10.1177/026988110201600304 12236627

[49] Thaler KJ Morgan LC Van Noord M Gaynes BN Hansen RA Lux LJ. Comparative effectiveness of second-generation antidepressants for accompanying anxiety, insomnia, and pain in depressed patients: a systematic review. Depress Anxiety. (2012) 29:495–505. 10.1002/da.21951 22553134

[50] Srinivasan V Zakaria R Othman Z Lauterbach EC Acuña-Castroviejo D. Agomelatine in depressive disorders: its novel mechanisms of action. J Neuropsychiatry Clin Neurosci. (2012) 24:290–308. 10.1176/appi.neuropsych.11090216 23037643

[51] Soumier A Banasr M Lortet S Masmejean F Bernard N Kerkerian-Le-Goff L. Mechanisms contributing to the phase-dependent regulation of neurogenesis by the novel antidepressant, agomelatine, in the adult rat hippocampus. Neuropsychopharmacology. (2009) 34:2390–403. 10.1038/npp.2009.72 19571795

[52] Engin E Treit D. The role of hippocampus in anxiety: intracerebral infusion studies. Behav Pharmacol. (2007) 18:365–74. 10.1097/FBP.0b013e3282de7929 17762507

[53] Fanselow MS Dong HW. Are the dorsal and ventral hippocampus functionally distinct structures? Neuron. (2010) 65:7–19. 10.1016/j.neuron.2009.11.031 20152109 PMC2822727

[54] Geugies H Mocking RJT Figueroa CA Groot PFC Marsman JC Servaas MN. Impaired reward-related learning signals in remitted unmedicated patients with recurrent depression. Brain. (2019) 142:2510–22. 10.1093/brain/awz167 31280309 PMC6734943

[55] Khazanov GK Xu C Dunn BD Cohen ZD DeRubeis RJ Hollon SD. Distress and anhedonia as predictors of depression treatment outcome: a secondary analysis of a randomized clinical trial. Behav Res Ther. (2020) 125:103507. 10.1016/j.brat.2019.103507 31896529 PMC6983353

[56] Di Giannantonio M Di Iorio G Guglielmo R De Berardis D Conti CM Acciavatti T. Major depressive disorder, anhedonia and agomelatine: an open-label study. J Biol Regul Homeost Agents. (2011) 25:109–14. 21382280

[57] Martinotti G Sepede G Gambi F Di Iorio G De Berardis D Di Nicola M. Agomelatine vs. venlafaxine XR in the treatment of anhedonia in major depressive disorder: a pilot study. J Clin Psychopharmacol. (2012) 32:487–91. 10.1097/JCP.0b013e31825d6c25 22722509

[58] Norman TR Olver JS. Agomelatine for depression: expanding the horizons? Exp Opin Pharmacother. (2019) 20:647–56. 10.1080/14656566.2019.1574747 30759026

[59] Lôo H Hale A D'Haenen H. Determination of the dose of agomelatine, a melatoninergic agonist and selective 5-HT(2C) antagonist, in the treatment of major depressive disorder: a placebo-controlled dose range study. Int Clin Psychopharmacol. (2002) 17:239–47. 10.1097/00004850-200209000-00004 12177586

[60] Kennedy SH Rizvi S Fulton K Rasmussen J. A double-blind comparison of sexual functioning, antidepressant efficacy, and tolerability between agomelatine and venlafaxine XR. J Clin Psychopharmacol. (2008) 28:329–33. 10.1097/JCP.0b013e318172b48c 18480691

[61] Montejo A Majadas S Rizvi SJ Kennedy SH. The effects of agomelatine on sexual function in depressed patients and healthy volunteers. Hum Psychopharmacol. (2011) 26:537–42. 10.1002/hup.1243 22102540

[62] Montejo AL Llorca G Izquierdo JA Rico-Villademoros F. Incidence of sexual dysfunction associated with antidepressant agents: a prospective multicenter study of 1,022 outpatients. Spanish working group for the study of psychotropic-related sexual dysfunction. J Clin Psychiatry. (2001) 62:10–21. 11229449

[63] Montejo A. L. Prieto N. Terleira A. Matias J. Alonso S. Paniagua G.. (2010). Better sexual acceptability of agomelatine (25 and 50 mg) compared with paroxetine (20 mg) in healthy male volunteers. An 8-week, placebo-controlled study using the PRSEXDQ-SALSEX scale. J Psychopharmacol. 24:111–120. 10.1177/0269881108096507 18801825

[64] Huang KL Lu WC Wang YY Hu GC Lu CH Lee WY. Comparison of agomelatine and selective serotonin reuptake inhibitors/serotonin-norepinephrine reuptake inhibitors in major depressive disorder: a meta-analysis of head-to-head randomized clinical trials. Aust N Z J Psychiatry. (2014) 48:663–71. 10.1177/0004867414525837 24604920

[65] Mi WF Tabarak S Wang L Zhang SZ Lin X Du LT. Effects of agomelatine and mirtazapine on sleep disturbances in major depressive disorder: evidence from polysomnographic and resting-state functional connectivity analyses. Sleep. (2020) 43:1144. 10.1093/sleep/zsaa092 32406918

[66] Tchekalarova J Kortenska L Ivanova N Atanasova M Marinov P. Agomelatine treatment corrects impaired sleep-wake cycle and sleep architecture and increases MT(1) receptor as well as BDNF expression in the hippocampus during the subjective light phase of rats exposed to chronic constant light. Psychopharmacology. (2020) 237:503–18. 10.1007/s00213-019-05385-y 31720718

[67] Kennedy SH Rizvi SJ. Agomelatine in the treatment of major depressive disorder: potential for clinical effectiveness. CNS Drugs. (2010) 24:479–99. 10.2165/11534420-000000000-00000 20192279

